# Frenzy

**Published:** 1867-07

**Authors:** 


### FRENZY.

"'ARE you ready for me! have you got the money?' and he went on heaping on me the most bitter taunts and opprobrious epithets; while speaking, he drew a handful of papers from his pockets, saying: 'I got you into your office, and now I'll get you out.' I can not tell how long these threats and invectives lasted. At first, I kept interposing, trying to pacify him. But I could not stop him. Soon, my own temper was up. I forgot every thing but the sting of his words. I was excited to the highest degree of passion; and in my fury I seized a small stick of wood and dealt him an instantaneous blow, with all the force that passion could give it. I did not know or think or care where nor how hard I should strike, nor what would be the effect. He fell instantly dead! I then cut up his body, hid a portion of it, and burned the remainder in a furnace." This was the confession of a highly educated man, just before he suffered the ignominious penalty of murder; the murder of the best friend he had on earth! It was done in an ecstacy of passion, in a "phrenzy," from a Greek word *phrene*, which means the mind; or a state of the brain in which the mind is excited to a pitch which places it beyond all human control; it is a moment-ary madness. The lesson sought to be impressed by this narration, is the danger of cherishing any mental excitement; and the consequent duty of studying how, in all possible ways, to keep the mental faculties in a uniformly calm, quiet, and deliberate condition. In the incident above, it was proven that half an hour before, the murderer had closed a philosophic lecture; and as he stepped from the rostrum into his own room, was met as above detailed, by a rich, remorseless creditor. In a very few minutes the calm philosopher was transformed into an ungovernable fury, by the utterance of a dozen taunting words; and had no more control over himself than an infant over an already sped thunderbolt. Cases are given in standard medical works, where the mental excitement has reached such an intensity, that the individual has fallen dead on the instant; even greater calamities are recorded; the loss of the mind forever, and the hapless victim has raved and raged in impotency behind the bars of a maniac's cell for the remainder of a long life; a fate surely worse than death! Sometimes the mind has gone out in eternal night with a fearful screech, combining the yell of the savage with the expressions of a demoniac.

Lesser degrees of mental excitement have found vent in words and manner so expressive, as to excite an uncontrollable horror in the minds of some of the hearers, and wilted the hearts of others, to bud and bloom no more. A single word uttered by a child to a parent, in a moment of excitement; of a parent to a child; of a husband to a wife, has many a time, before now, quenched every spark of human emotion and of human love, and a hate has sprung from the ashes, as virulent as the deadly upas, only to go out in the night of the grave. Human happiness, and life itself, then, often depends on a failure to control the mental emotion. An effort to practice such a control should be early made; the earlier. the better. And let it be particularly remembered, that the most effectual practical manner of doing this, is to cultivate a habit of speaking in a low, slow, deliberate tone of voice, under all circumstances; but whenever the circumstances are exciting, speak not a syllable until the thought, embodied in words, stands out plainly before the mind, "My God and Father is here," and then speak accordingly. The reason of this lies in the curious fact, that the mind has a faculty of being persuaded to believe what the lips express, although every word is a falsehood; for in the excited condition, that which is called imagination runs riot, and makes the merest presumption appear for a moment to be an actual fact. This is an every day occurrence in domestic life, where an excited husband or wife begins to talk of a supposed insult, or deviation of a servant; and the more they talk, the greater appears the aggravation. Reader, keep ever before you the fear of "frenzy," for in an unguarded hour, within any dozen minutes, it may lead you to utter a word against a heart that loves you, whose wound no tears can ever wash away; may lead you to commit an act which will send you to the gallows or a mad-house!

